# Open complete dislocation of trapezium with a vertically split fracture: a case report

**DOI:** 10.1186/1757-1626-2-9092

**Published:** 2009-11-26

**Authors:** Mohammad Umar Mumtaz, Najeeb Ahmad Drabu

**Affiliations:** 1 Department of Orthopaedics, Government JLN Memorial Hospital, Srinagar, Kashmir, India

## Abstract

Open complete dislocation of the trapezium is an extraordinarily rare injury with only a few cases reported so far in literature. The association of a vertically split fracture makes this injury even rare and hence worth reporting. A 14 year old Kashmiri boy presented to us with a history of massive trauma to the non dominant left hand sustained as a result of a blow from a heavy hammer. The thenar area was burst out and the trapezium was vertically split apart into two halves which were dislocated from the articular surfaces of the scaphoid as well as the first metacarpal. The mechanism of injury as in other such reported cases was a massive direct force localized over the carpal bone which causes its enucleation and fracture. Although some authors have recommended excision of the dislocated trapezium, open reduction of the fracture dislocation and fixation with K wires was carried out under General anesthesia. At the end of one year although there was some functional deficit in the affected thumb, especially in opposition, the patient was quite satisfied with the outcome as this was the non dominant hand.

## Introduction

Fractures of the trapezium account for 3-5% of carpal bone fractures and Less than a fifth of these are sagitally split[1]. Open dislocation of trapezium at first carpometacarpal and scaphotrapezial joints is a very rare injury [2,3]. After review of literature Peterson [4] in1950 reported only 10 cases of trapezium dislocation, however only two of these could be considered as complete dislocations. Afterwards a few more of such cases have been described[2,3]. We report an extraordinarily rare combination of open complete dislocation of Trapezium at first carpometacarpal and scapho trapezial joints associated with a vertically split open book type fracture.

## Case Presentation

**Figure 1:**
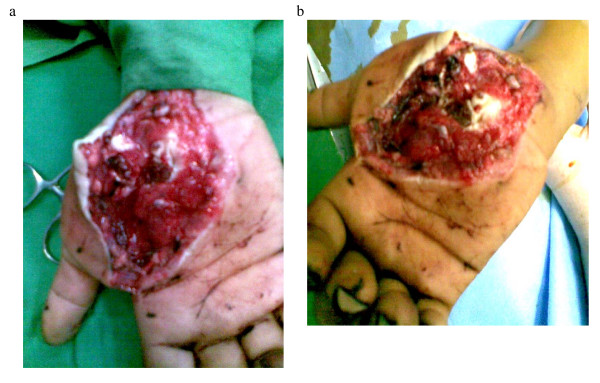
**Photographs of the case showing a burst open injury of the thenar aspect of palm**. The trapezium is split apart sagitally into two halves which are also dislocated from the articular surfaces of the first metacarpal base and the scaphoid (clearly exposed in the photographs)

**Figure 2:**
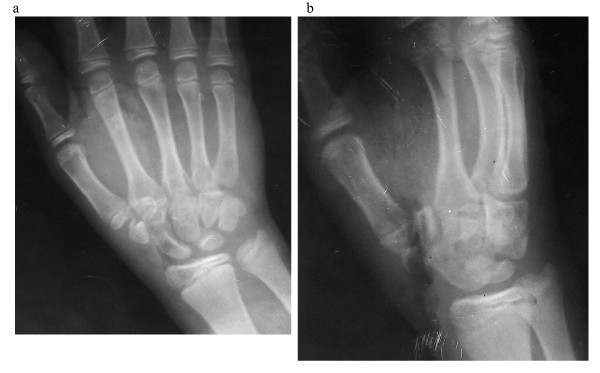
**Radiographs of the above patient before surgery**.

**Figure 3:**
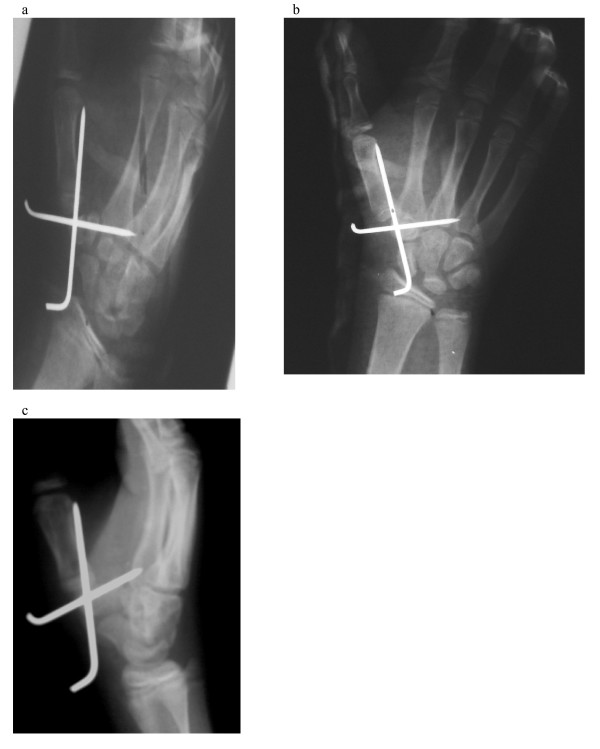
**Radiographs of the same patient after open reduction of the fracture dislocation and stabilization with K wires**.

A fourteen year old Kashmiri boy was admitted to the emergency department of our hospital with injury to his non dominant left hand. The patient was assisting his father, a blacksmith in their workshop, when accidentally his hand was struck on the dorsum with a severe blow from a heavy hammer. The massive impact of the hammer on the dorsum coupled with a counter force from the edge of the iron base on which he was holding some object, caused the thenar area to burst open and at the same time produced a sagitally split fracture of the trapezium with dislocaton at first carpometacarpal and scapho trapezial joints. On examination there was a huge, gaping lacerated wound extending from the web space of the thumb upto 2 cm above the flexion crease of the wrist [Fig 1a and 1b]. The thenar muscles were divided and the trapezium was vertically split apart into two halves which were dislocated from the articular surfaces of the scaphoid as well as the first metacarpal with rupture of the corresponding ligaments. The injury gave an an open book appearance [fig. 1a and 1b]. There was no function of thenar muscles. Sensation of thumb was preserved. The FPL was intact. The digital vessels of thumb were kinked but intact and distal capillary refill was sluggish compared to other digits. Radiological examination confirmed the clinical findings of vertical split trapezial fracture with dislocation at first carpometacarpal and scapho trapezial joints [fig 2a and 2b]. Exploration was carried under GA and tourniquet. Thorough wound irrigation and debridement was done. The cancellous surfaces of the two halves of trapezium were curetted and the fracture reduced with a K wire. The trapezium was next reduced back to its articulations with the first metacarpal and scaphoid. A second K wire was used to stabilize the trapezium to first metacarpal base [fig 3a, 3b and 3c]. The thenar muscles were repaired and wound closed loosely over a glove drain. A dorsal plaster slab was applied Intraoperative imaging facilities were not available in this emergency setup but post operative roentgenograms revealed that the K wire fixing the trapezium to the first metacarpal had little purchase in the base of the metacarpal (figs 3A-3C), however we decided against disturbing this arrangment because the alignment was maintained and reinforcement with a plaster splint was considered adequate. Besides reinsertion of this wire would have meant another anesthesia. Wound dressings were carried out through a window. The circulation (Capillary refill) improved progressively over the next few days. The K wires and plaster slab were removed after three weeks and a removable splint was applied. An intensive physiotherapy program was started. After 8 weeks the splint was discarded. At the end of one year there was a gross deficit in opposition with a moderate deficit in adduction which was most probably a result attributed to soft tissue scarring involving the thenar muscles. However abduction and extension were only slightly impaired. Despite these deficits, the patient was quite satisfied with the outcome as this was the non dominant hand.

## Discussion

Open dislocation of trapezium at first carpometacarpal and scaphotrapezial joints is a very rare injury[2,3]. Further an associated fracture of trapezium is an extremely rare injury. After review of literature Peterson in 1950 reported only 10 cases of trapezium dislocation, however only two of these could be considered as complete dislocations. Afterwards Siegel [2] described a case of compound complete dislocation of trapezium with a communited fracture of the trapezoid. Later Seimon [3] also described another case of open complete dislocation of trapezium with a fracture of the ridge of trapezium. The ligaments binding trapezium to the first metacarpal and the scaphoid are very Strong [2]. Severe direct trauma is necessary to produce the disruption of these ligaments and hence a complete dislocation [2,3]. This surely was the case in our patient. A review of other reported cases also reveals a similar mechanism of injury in all cases, a massive direct force localized over the carpal bone which causes its enucleation. Some authors [4] recommended excision of the dislocated trapezium. In the case of our the patient open reduction and K wire fixation yielded satisfactory results.

## Consent

Written informed consent for publication of this case report and accompanying images was obtained from the father of the patient as he is a minor.

## Competing interests

The authors declare that they have no competing interests.

## Authors' contributions

Both the authors have a major contribution in this case report. Both were involved in the pre operative planning, surgery, post operative management as well as in the preparation of this manuscript.
